# Supplementary material for: Phenotypic evolution of SARS-CoV-2 spike during the COVID-19 pandemic
Source: Nat Microbiol. 2025 Jan 3;10(1):77–93. doi: 10.1038/s41564-024-01878-5 (PMC11726466; doi:10.1038/s41564-024-01878-5)
Supplement: Supplementary file 1 — Reporting Summary [file 41564_2024_1878_MOESM1_ESM.pdf]

nature portfolio

Corresponding author(s): Massimo Palmarini
Arvind Patel

Last updated by author(s): Nov 6, 2024

Reporting Summary

Nature Portfolio wishes to improve the reproducibility of the work that we publish. This form provides structure for consistency and transparency in reporting. For further information on Nature Portfolio policies, see our Editorial Policies and the Editorial Policy Checklist.

Statistics

For all statistical analyses, confirm that the following items are present in the figure legend, table legend, main text, or Methods section.

n/a	Confirmed	
☐	☒	The exact sample size (*n*) for each experimental group/condition, given as a discrete number and unit of measurement
☐	☒	A statement on whether measurements were taken from distinct samples or whether the same sample was measured repeatedly
☐	☒	The statistical test(s) used AND whether they are one- or two-sided<br>*Only common tests should be described solely by name; describe more complex techniques in the Methods section.*
☐	☒	A description of all covariates tested
☐	☒	A description of any assumptions or corrections, such as tests of normality and adjustment for multiple comparisons
☐	☒	A full description of the statistical parameters including central tendency (e.g. means) or other basic estimates (e.g. regression coefficient) AND variation (e.g. standard deviation) or associated estimates of uncertainty (e.g. confidence intervals)
☐	☒	For null hypothesis testing, the test statistic (e.g. *F*, *t*, *r*) with confidence intervals, effect sizes, degrees of freedom and *P* value noted<br>*Give P values as exact values whenever suitable.*
☒	☐	For Bayesian analysis, information on the choice of priors and Markov chain Monte Carlo settings
☒	☐	For hierarchical and complex designs, identification of the appropriate level for tests and full reporting of outcomes
☒	☐	Estimates of effect sizes (e.g. Cohen's *d*, Pearson's *r*), indicating how they were calculated

Our web collection on statistics for biologists contains articles on many of the points above.

Software and code

Policy information about availability of computer code

Data collection	Odissey infrared CLx Imager with Image Studio Lite software V 5.2, Applied Biosystems 7500 software, CLARIOstar Plus microplate reader (BMG Labtech), GloMax Explorer GM3500 multimode plate reader (Promega), Aperio VERSA Pathology slide scanner with software V1.0.1.125 and ImageScope V12.4.3 (Leica)
Data analysis	Applied Biosystems 7500 software, Image Studio Lite V5.2 (LI-COR), MARS data analysis software v5.02 R1 (BMG Labtech), GraphPad Prism Versions 9 & 10.2.2, HALO Image analysis platform V3.6 (Indica Labs), QuPath digital pathology and whole slide image analysis software V0.3.2, UCSF ChimeraX (UCSF RBVI)

For manuscripts utilizing custom algorithms or software that are central to the research but not yet described in published literature, software must be made available to editors and reviewers. We strongly encourage code deposition in a community repository (e.g. GitHub). See the Nature Portfolio guidelines for submitting code & software for further information.

Data

Policy information about availability of data

All manuscripts must include a data availability statement. This statement should provide the following information, where applicable:

- Accession codes, unique identifiers, or web links for publicly available datasets
- A description of any restrictions on data availability
- For clinical datasets or third party data, please ensure that the statement adheres to our policy

All data generated and analysed during this study are included in this manuscript and supplementary information files will be made available before publication. SARS-CoV-2 sequence counts and genomic data were extracted from GISAID and COG-UK. No new algorithms were developed for this project.Raw data underpinning the figuresfigures associated with this manuscript are available in the Enlighten repository (https://doi.org/10.5525/gla.researchdata.1698)

Research involving human participants, their data, or biological material

Policy information about studies with human participants or human data. See also policy information about sex, gender (identity/presentation), and sexual orientation and race, ethnicity and racism.

Reporting on sex and gender	N/A
Reporting on race, ethnicity, or other socially relevant groupings	N/A
Population characteristics	N/A
Recruitment	N/A
Ethics oversight	N/A

Note that full information on the approval of the study protocol must also be provided in the manuscript.

Field-specific reporting

Please select the one below that is the best fit for your research. If you are not sure, read the appropriate sections before making your selection.

☒ Life sciences ☐ Behavioural & social sciences ☐ Ecological, evolutionary & environmental sciences

For a reference copy of the document with all sections, see nature.com/documents/nr-reporting-summary-flat.pdf

Life sciences study design

All studies must disclose on these points even when the disclosure is negative.

Sample size	No statistical methods were used to pre-determine sample sizes. Our sample sizes are similar to those reported in previous publications (Willett et al, 2022, Nature Microbiology; Reuschl et al, 2024, Nature Microbiology; Meehan et al, 2023, Plos Pathogens; Meng et al, 2022, Nature; Peacock et al, 2021, Nature Microbiology. Multiple independent experiments were repeated to allow for appropriate statistical analysis.
Data exclusions	No data were excluded
Replication	In vitro experiments were performed independently at least 3 times (unless stated otherwise) to allow for appropriate confidence in the reproducibility of the results. All attempts at replication were successful.
Randomization	No randomisation was performed. Experimental groups were treated identical except for the specific variables being tested. Thus, randomisation is not required.
Blinding	Blinding was not necessary as all measurements were quantified by automated machines and softwares. No data were excluded.

Reporting for specific materials, systems and methods

We require information from authors about some types of materials, experimental systems and methods used in many studies. Here, indicate whether each material, system or method listed is relevant to your study. If you are not sure if a list item applies to your research, read the appropriate section before selecting a response.

Materials & experimental systems

n/a	Involved in the study
☐	☒ Antibodies
☐	☒ Eukaryotic cell lines
☒	☐ Palaeontology and archaeology
☐	☒ Animals and other organisms
☒	☐ Clinical data
☒	☐ Dual use research of concern
☒	☐ Plants

Methods

n/a	Involved in the study
☒	☐ ChIP-seq
☒	☐ Flow cytometry
☒	☐ MRI-based neuroimaging

Antibodies

Antibodies used

Primary antibodies for western blot:
Rabbit anti-SARS-CoV-2 spike S2 (ThermoFisher, PA1-41165), sheep anti-SARS-CoV-2 nucleocapsid protein DA114(Rihn et al, 2021, Plos Biology https://mrcppu-covid.bio/antibodies/134473), rabbit anti-phospho STAT1 Tyr701 clone 58D6 (CellSignaling Technologies, 9167), mouse anti-alpha-Tubulin DM1A (Sigma-Aldrich, T6199), rabbit anti-RSAD2 (Proteintech, 28089-1-AP), mouse anti-IFIT1 (Origene, TA5009487). All used at 1:1000 dilution.

Secondary antibodies for western blot:
anti-rabbit IgG (H+L) DyLight 800 conjugate (CellSignalling Technology, 5151S, 1:20000 dilution), anti-mouse IgG (H+L) DyLight 680 conjugate (CellSignalling Technology, 5470S, 1:15000 dilution), anti-sheep IgG451 (H+L) DyLight 800 (Thermo Fisher, SA5-10060, 1:15000 dilution).

Primary antibodies for immunohistochemistry:
rabbit anti-IBA-1 (Alpha labs, 019-19741, 1:2500 dilution), mouse anti-TTF1 (Leica Biosystems, NCL-L-TTF-1, 1:200 dilution), rabbit anti-h/m Active Caspase 3 (R&D systems, AF835, 1:500 dilution), CD3 (Agilent Dako, A0452, 1:200 dilution).

Validation

Rabbit anti-SARS-CoV-2 spike S2 (ThermoFisher, PA1-41165), validated by the manufacturer: https://www.thermofisher.com/antibody/product/SARS-Coronavirus-Spike-Protein-Antibody-Polyclonal/PA1-41165
Sheep anti-SARS-CoV-2 nucleocapsid protein (Rihn et al, 2021, Plos Biology): Validated by comparison of mock-infected and infected cell lysates. Migrates at the expected molecular weight (Rihn et al, 2021, Plos Biology https://doi.org/10.1371/journal.pbio.3001091)
Rabbit anti-phospho STAT1 Tyr701 clone 58D6 (CellSignaling Technologies, 9167): Validated by the manufacturer. Detects endogenous levels of STAT1 only when phosphorylated at tyrosine 701. It does not cross-react with the corresponding phospho-tyrosines of other STAT proteins. https://www.cellsignal.com/products/primary-antibodies/phospho-stat1-tyr701-58d6-rabbit-mab/9167
mouse anti-alpha-Tubulin DM1A (Sigma-Aldrich, T6199): Validated by the manufacturer: https://www.sigmaaldrich.com/GB/en/product/sigma/t6199
rabbit anti-RSAD2 (Proteintech, 28089-1-AP), validated by the manufacturer: https://www.ptglab.com/products/RSAD2-Antibody-28089-1-AP.htm
mouse anti-IFIT1 (Origene, TA5009487), validated by the manufacturer: https://www.origene.com/catalog/antibodies/primary-antibodies/ta500948/ifit1-mouse-monoclonal-antibody-clone-id-oti3g8

rabbit anti-IBA-1 (Alpha labs, 019-19741), validated by the manufacturer https://www.alphalabs.co.uk/019-19741#literature
mouse anti-TTF1 (Leica Biosystems, NCL-L-TTF-1), validated by the manufacturer: https://shop.leicabiosystems.com/en-gb/ihc-ish/ihc-primary-antibodies/pid-thyroid-transcription-factor-1.
rabbit anti-h/m Active Caspase 3 (R&D systems, AF835), https://www.rndsystems.com/products/human-mouse-active-caspase-3-antibody_af835. CD3 (Agilent Dako, A0452), validated by the manufacturer, https://www.agilent.com/en/product/immunohistochemistry/antibodies-controls/primary-antibodies/cd3-(concentrate)-76133.

Eukaryotic cell lines

Policy information about cell lines and Sex and Gender in Research

Cell line source(s)

Calu-3 cells were commercially obtained from ATCC (HTB-55), A549 cells (ATCC, #CCL-185) expressing hACE2 and TMPRSS2 were generated at the CVR and described previosuly (Rihn et al, 2021, Plos Biology). HEK-293T cells (ATCC, #CRL-3216) expressing hACE2 were described previously (Willett et al, 2022, Nature Microbiology). Reconstituted human nasal and bronchial epithelium cultures hNECS and hBECs were commercially obtained from EPITHELIX, Switzerland (Peacock et al, 2021, Nature Microbiology; Willett et al, 2022, Nature Microbiology). BHK-N-hACE2 cells deriving from BHK-21 () ATCC #CCL-10 were generated at CVR and described previously (Willett et al, 2022, Nature Microbiology).

Authentication

Cell lines were commercially procured and authenticated by the supplier.

Mycoplasma contamination

Mycoplasma testing was conducted throughout the duration of the study and cells tested negative.

Commonly misidentified lines
(See ICLAC register)

None

Animals and other research organisms

Policy information about studies involving animals; ARRIVE guidelines recommended for reporting animal research, and Sex and Gender in Research

Laboratory animals	8-12 weeks old Golden syrian hamsters (HsdHan®:AURA)
Wild animals	No wild animals were used in the study
Reporting on sex	All experiments were done with sex and age matched groups. Bot sexes were used equally split within each group.
Field-collected samples	No field collected samples were used in the study
Ethics oversight	Procedures were performed under UK Home Office Licence PP0271643 in accordance with the Animals (Scientific Procedures) Act 1986 and approved by the University of Glasgow ethics committee. All animal research adhered to ARRIVE guidelines.

Note that full information on the approval of the study protocol must also be provided in the manuscript.

Plants

Seed stocks	*Report on the source of all seed stocks or other plant material used. If applicable, state the seed stock centre and catalogue number. If plant specimens were collected from the field, describe the collection location, date and sampling procedures.*
Novel plant genotypes	*Describe the methods by which all novel plant genotypes were produced. This includes those generated by transgenic approaches, gene editing, chemical/radiation-based mutagenesis and hybridization. For transgenic lines, describe the transformation method, the number of independent lines analyzed and the generation upon which experiments were performed. For gene-edited lines, describe the editor used, the endogenous sequence targeted for editing, the targeting guide RNA sequence (if applicable) and how the editor was applied.*
Authentication	*Describe any authentication procedures for each seed stock used or novel genotype generated. Describe any experiments used to assess the effect of a mutation and, where applicable, how potential secondary effects (e.g. second site T-DNA insertions, mosiacism, off-target gene editing) were examined.*
