# Supplementary material for: Phenotypic evolution of SARS-CoV-2 spike during the COVID-19 pandemic
Source: Nat Microbiol. 2025 Jan 3;10(1):77–93. doi: 10.1038/s41564-024-01878-5 (PMC11726466; doi:10.1038/s41564-024-01878-5)
Supplement: Supplementary file 11 — Unprocessed western blots. [file 41564_2024_1878_MOESM11_ESM.pdf]

Raw blots images used in Extended Data Figure 3a

4-12% Bis-Tris gels ran in MOPS running buffer

Whole membrane probed 1st for Spike then probed for N protein

Cropped area for figure

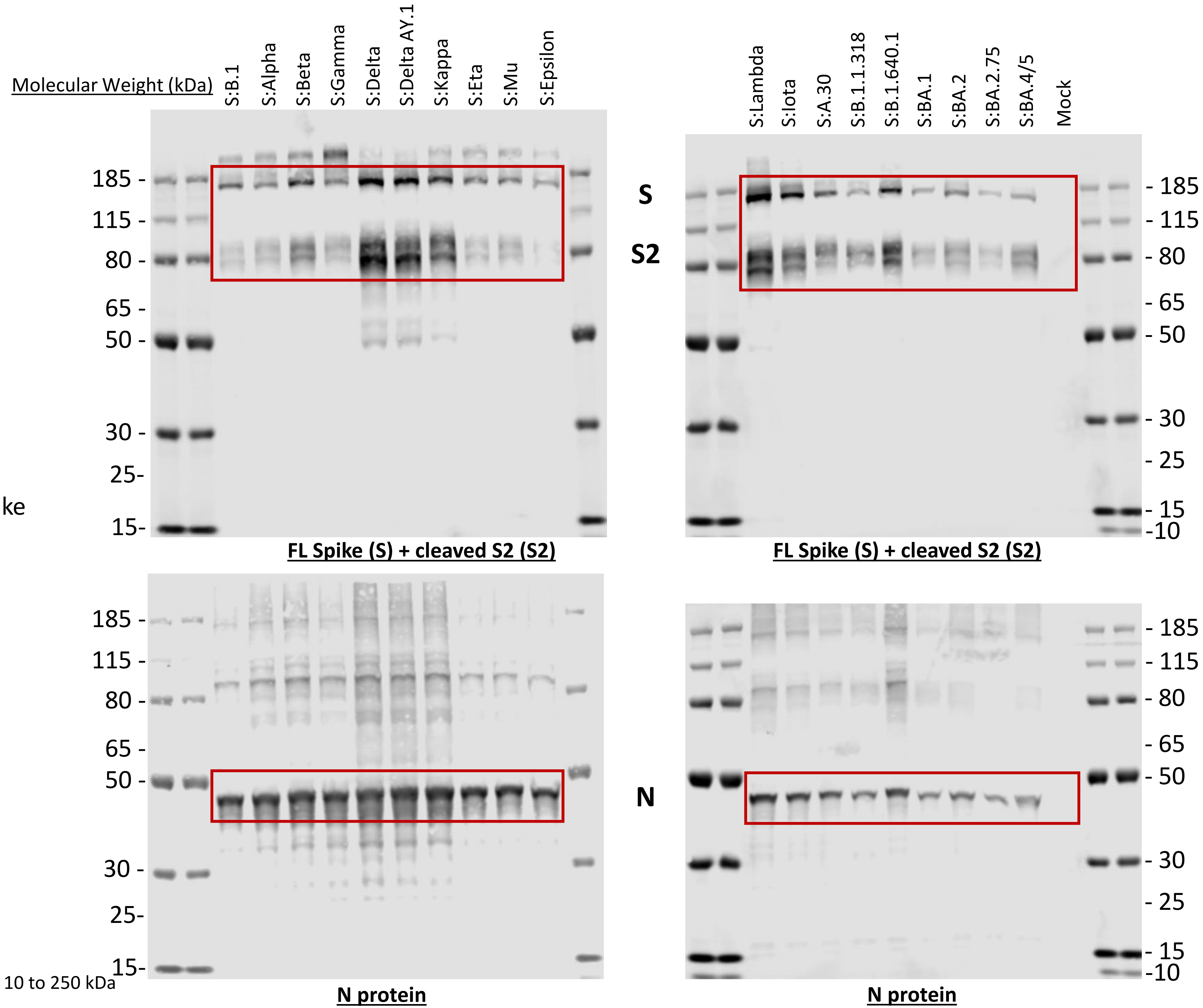

Thermo Scientific™ PageRuler™ Plus Prestained Protein Ladder, 10 to 250 kDa
